# Alliance Between Therapist and Multi-stressed Families During the COVID-19 Pandemic: The Effect of Family-Based Videoconferencing

**DOI:** 10.1007/s10566-021-09644-y

**Published:** 2021-08-17

**Authors:** Aurelie. M. C. Lange, Marc J. M. H. Delsing, Marieke van Geffen, Ron. H. J. Scholte

**Affiliations:** 1 Department of Psychiatry, Section of Medical Psychology & Psychotherapy, Erasmus Medical Center, Rotterdam, The Netherlands; 2 Viersprong Institute for Studies on Personality Disorders, Halsteren, The Netherlands; 3 Praktikon, Nijmegen, The Netherlands; 4 Behavioural Science Institute, Radboud University, Nijmegen, The Netherlands; 5 Tranzo, Tilburg University, Tilburg, The Netherlands

**Keywords:** Therapeutic alliance, Covid-19, Videoconferencing, Systemic therapy, Multi-stressed families

## Abstract

**Background:**

A strong therapeutic alliance or working relationship is essential for effective face-to-face family-based psychotherapy. However, little is known about the use of VC on alliance in family-based therapy. The recent COVID-19 pandemic led to a national lockdown during which most family-based therapy transferred to VC.

**Objective:**

The current study analyzed the development and strength of alliance prior and during lockdown for multi-stressed families participating in Multisystemic Therapy (MST).

**Method:**

Alliance with the therapist was reported monthly by 846 caregivers (81% female). Using latent growth curve models (longitudinal approach), the development of alliance was estimated for families participating in MST prior to the lockdown, transferring to VC early in treatment or late in treatment. Using regression analyses (cross-sectional approach), lockdown (yes/no) was included as predictor of alliance. In these analyses, type of family (regular; intellectual disability; concerns regarding child abuse or neglect) and gender of caregiver were included as moderators.

**Results:**

Both analytical approaches showed that alliance was not affected by VC, except for families with concerns of child abuse, who reported lower alliances during lockdown. However, these results where no longer significant when controlling for multiple testing.

**Conclusions:**

Strong alliances can be developed and maintained during family-based VC sessions with multi-stressed families. However, for some subgroups, such as families with concerns of child abuse, VC might not be suitable or sufficient. Future research needs to investigate the potential and limitations of using VC with families.

## Introduction

### Alliance

Research suggests that the therapeutic alliance, or the working relationship between a client and the therapist, is an important aspect of effective psychotherapeutic interventions (Flückiger et al., 2020a). Alliance consists of the affective and collaborative aspects of a client–therapist relationship and is usually conceptualized as personal alliance (the affective bond) and task-related alliance (addressing the goals of the treatment and the tasks required to achieve those goals; Bordin, 1979; Hougaard, 1994). A strong alliance is associated with positive treatment outcomes and higher client satisfaction in a wide range of psychotherapeutic interventions, such as individual child, adolescent and adult psychotherapy, family-based or systemic therapy, and parenting interventions (de Greef et al., 2017; Friedlander et al., 2018; Karver et al., 2018; Welmers-van de Poll et al., 2018).

Forming strong alliances with families with multiple or complex problems, often termed as multi-problem or multi-stressed families, may be especially challenging (Escudero & Friedlander, 2017). These families struggle with a range of problems—such as parental psychosocial problems, parenting issues, child psychosocial problems, and socio-economic problems—and have often been involved with numerous care professionals for a number of years. As a consequence, multi-stressed families may mistrust new professionals, lack motivation for additional treatments, and may also have given up hope of any successful outcome from new interventions (Deković & Bodden, 2019; Escudero & Friedlander, 2017). Research suggests that it may be challenging to create a strong therapeutic alliance with these families, especially when treatment is involuntary (Escudero & Friedlander, 2017; McLeod et al., 2016; Sotero et al., 2016). Family members may have different opinions about the most pressing problems and the goals for the intervention. They may further feel unsafe when court or child protective services are involved (Escudero & Friedlander, 2017; Sotero et al., 2016). To build a strong therapeutic alliance and subsequently work towards positive outcomes with these families, professionals providing family-based interventions usually visit the family several times a week, often in their home environment. Moreover, professionals actively and intensively work on developing and maintaining a strong alliance during the whole intervention period (Visscher et al., 2020).

### COVID-19 Pandemic

In early 2020, a new virus (COVID-19) spread rapidly across the world causing severe respiratory problems for those infected. To contain this virus and decrease the infection rate, the Dutch government announced a national lockdown in March 2020. Schools were closed and it was strongly advised to stay at home and work from home. This meant that some parents had to combine working from home with home schooling their children, while others lost their job due to the forced closure of multiple industries, such as the catering industry and the cultural sector. Preliminary research suggests these hardships have increased parental stress and parent mental health problems (Creasey et al., 2021; Gassman-Pines et al., 2020; Whittle et al., 2020) although some families may have benefited from the additional time spent together and the decrease of external demands and stressors, leading to more warmth and less conflicts (Bülow et al., 2020; Spanje-Hennes & Kesselring, 2020).

The Dutch organization of mental health and addiction care providers in the Netherlands developed a guidance on how to continue providing mental health care in a safe way while also complying with the new national rules announced by the government. The advice was to deliver care remotely (through phone or video) whenever possible (GGZ Standaarden, 2020). Although there were some differences between mental health institutions regarding how they applied this guidance, in general, intensive home-based interventions were transformed into therapy through videoconferencing. Additionally, inventive solutions for contact, such as walks in the park or window-sessions, have been used (Steketee et al., 2020). The transition to remote therapy may have had substantial consequences for the way in which, and the success with which, professionals could build alliances with families. The current study aimed to investigate the effect of videoconferencing during the COVID-19 pandemic on the therapeutic alliance between professionals and families.

### Videoconferencing

The pandemic fueled an unpreceded transfer to remote therapy means, such as videoconferencing. Never before has videoconferencing, or telepsychotherapy in general, been used in so many treatment settings with so many clients. Yet, telepsychotherapy is not a new phenomenon and has been studied for over 20 years (Backhaus et al., 2012). There are many different types of telepsychotherapy, ranging from self-guided therapy without therapist contact to fully therapist-guided therapy through videoconferencing. Moreover, telepsychotherapy can either be fully online or combined with face-to-face (F2F) contact, called blended therapy (Berger, 2017). We will discuss therapist-led videoconferencing (VC), as VC seems to have been the most-used remote treatment form during the COVID-19 pandemic.

Overall, most research on VC has focused on individual psychotherapy (often cognitive behavioral therapy) with adults. Less is known about VC for family-based treatments, involving multiple family members in the same session. Studies on multi-stressed families are even sparser. Reviews and meta-analyses focusing primarily on individual therapy with adult populations suggest that VC can achieve comparable outcomes as standard F2F psychotherapy (Backhaus et al., 2012; Norwood et al., 2018). A recent meta-analysis regarding VC for therapy with families and couples reports similar findings, suggesting that VC is feasible, acceptable and effective with couples and families (De Boer et al., 2021). Most studies in this study, however, focused on couples or families with young children. Few studies involved families with adolescents, especially families with high risks such as family violence (De Boer et al., 2021).

Regarding the strength of alliance in VC, results are somewhat mixed. Whereas two reviews suggest that alliance in VC is comparable to alliance in F2F therapy (Berger, 2017; Simpson & Reid, 2014), a meta-analysis found alliance to be inferior in VC (Norwood et al., 2018). This latter finding might have been due to the inclusion of therapist-reported alliance scores. Several studies suggest that therapists may report more negatively on the alliance in VC than in standard therapy, whereas client-reported alliance does not seem to be impacted by the medium (Berger, 2017; Norwood et al., 2018). An RCT by Watts et al. (2020), on the other hand, found no difference between therapist-reported alliance in VC and F2F therapy, whereas clients reported stronger alliances in VC than in F2F therapy. Multiwave studies on adults have shown that the development of alliance is comparable in VC and F2F therapy (Germain et al., 2010; Watts et al., 2020). The few studies focusing specifically on family-based interventions using VC report strong alliances (De Boer et al., 2021; Sibley et al., 2017) even if clients are initially skeptical about whether VC will be effective (Kysely et al., 2020). Only one study compared the family-based VC intervention to a F2F alternative. They did not find a significant difference in alliance between both groups (Comer et al., 2017).

Qualitative studies and descriptive papers further provide relevant information. For example, research suggests that very young children, older generations, clients lacking motivation for treatment or families with lower social economic status or education might experience more difficulty handling the required technology and/or may be more difficult to engage in VC sessions (Crum & Comer, 2016; Florean et al., 2020; Matheson et al., 2020). Some adolescents may further feel screenshy (Burgoyne & Cohn, 2020; Mc Kenny et al., 2021). Working with multi-stressed families may involve multiple of these challenges, as these families may be more likely to have a low social economic status, lack motivation, and treatment may involve working with the children or adolescents. Moreover, common challenges of VC are technology failures and interruptions by other family members (Sibley et al., 2017). Again, interruptions may be more frequent when interacting with multiple family members in the same session. At the same time, advantages have been mentioned as well; clients may experience an increased feeling of control, safety or empowerment and the flexibility of VC may improve session attendance (Mc Kenny et al., 2021; Sibley et al., 2017; Simpson & Reid, 2014). Thus, although several challenges can be hypothesized when using VC in a systemic context with multi-stressed families, more research is needed to understand the feasibility and effectiveness of family-based VC.

The current research included a large sample of multi-stressed families participating in a family-based intervention prior to or during the COVID-19 pandemic, thereby receiving F2F or VC treatment, and compared the strength and development of alliance across these two conditions.

### Videoconferencing During the COVID-19 Pandemic

Although there is a considerable amount of research on VC, the evidence regarding VC for family-based interventions is much smaller. Moreover, there are some important differences between previous research and the situation of the COVID-19 pandemic. First, most previous studies have focused on newly developed VC alternatives, provided by therapists who, most likely, felt comfortable using them. The COVID-19 pandemic, however, forced therapists to transition to VC very suddenly and often without appropriate training or preparation. A survey among 145 psychotherapists across North America and Europe showed that half of the sample did not have any experience with telepsychotherapy prior to the pandemic and only one third of the sample followed a training or webinar to prepare oneself for the transition to VC, although the majority of the therapists did talk to colleagues or read posts as preparation (Békés & Aafjes-van Doorn, 2020). Not surprisingly, therapists providing VC during the COVID-19 pandemic experienced more self-doubt and anxiety and felt less competent when using VC instead of F2F therapy. Moreover, these feelings of self-doubt and anxiety were negatively related to alliance (Aafjes-van Doorn et al., 2020). This suggests that alliance may have been negatively impacted by the pandemic. Second, research suggests that effective use of VC requires specific therapist behaviors, such as more frequent checks on client perspectives on the alliance, more verbal communication and more explicit use of non-verbal communication (Crum & Comer, 2016; Simpson & Reid, 2014). It may require some experience before a therapist can use these behaviors confidently and effectively. Many therapists may not have had such experience when they transitioned to VC during the pandemic. Third, informal communication with providers and coaches of family-based interventions suggests that therapists experienced considerable stress from the pandemic, both in their personal life (e.g., loneliness or stress from combining home-schooling with work) as well as professionally (e.g., having to adapt to delivering treatment differently and losing valuable F2F colleague contact). This additional stressor was not present in previous studies on VC and may have impacted their ability to develop strong alliances. A last difference between the situation investigated in previous research and the current situation is the extent to which clients could join in the decision making process. Research shows that shared decision making can improve client engagement in treatment (Metz, 2018). During the COVID-19 pandemic, however, the transition to VC was practically enforced. Given these differences, findings from previous research may not apply to alliance in VC during the COVID-19 pandemic.

### Current Study

The current study investigated how the strength and development of the alliance between the therapist and the primary caregiver is affected by the changes in delivery of care during to the first three months of the COVID-19 pandemic in the Netherlands. For this purpose, we used data collected as part of the quality assurance system of Multisystemic Therapy (MST). MST is an evidence-based intervention addressing all environmental systems (home, school, friends, and neighborhood) that impact 12- to 18-year-old adolescents with antisocial and/or delinquent behavioral problems. Families referred to MST can often be characterized as multi-stressed families, experiencing not only child behavioral problems, but equally parenting problems, parent psychosocial problems and/or socio-economic problems. MST is intensive (multiple sessions a week), short (three to five months) and home-based. Although therapists collaborate with all systems surrounding the adolescent, as well as work with the adolescent him-/herself where possible, MST therapists primarily work with parents and caregivers to reduce behavioral problems of the adolescent (Henggeler et al., 2009).

As part of the ongoing quality assurance system of MST, aimed at monitoring and upholding adherent delivery of MST, families are phoned by an independent call center on a monthly basis to complete a questionnaire regarding therapist adherence and alliance (Henggeler & Schoenwald, 1999).

The aim of this study was to investigate whether alliance was affected by using VC during the COVID-19 pandemic, using two approaches. Using a longitudinal approach, we assessed whether the trajectory of alliance during treatment was affected by the timing of the transfer to VC (after treatment; late in treatment; early in or prior to treatment). We also used a cross-sectional approach to investigate whether certain phases in treatment were more vulnerable for decreases in alliance due to VC than other phases. Moreover, we included two moderators, namely (1) type of family (families with an intellectual disability; families experiencing child abuse or neglect; regular families) and (2) gender of the primary caregiver (and hence alliance informant). Thus, we aimed to develop a more nuanced understanding for whom VC might be more or less suitable.

Building a strong alliance may be more challenging with certain families than with others. For example, communication with clients with an intellectual disability may require visual support and concrete and simple language. This impacts upon how therapists develop a therapeutic alliance and may be especially challenging through VC. Likewise, building alliances with caregivers who may be neglectful towards their children may be challenging. Research suggests that in such situations, caregivers may test professionals before accepting to build a working relationship with them (Reimer, 2013). We hypothesized that building trust with these clients may be even more challenging through VC than when therapists can go on home visits. To date, very little is known about the feasibility of using VC with these clients (Madhavan, 2019). Hence, we hypothesized these families to be at increased risk of experiencing adverse effects of the transfer to VC due to the COVID-19 pandemic on their alliances.

We were also interested in parental gender differences as research suggests that professional-parent alliances differ between fathers and mothers. This may be due to different dynamics between the parent and the professional, as well as differences in parenting style and parent–child relationships between fathers and mothers (de Greef et al., 2017). Moreover, the COVID-19 pandemic may impact men and women differently, which may also have influenced their alliances with professionals (Ausín et al., 2021; Connor et al., 2020; Czymara et al., 2020). For example, research suggests that women experienced more anxiety, depression and post-traumatic stress symptoms during the COVID-19 pandemic (Ausín et al., 2021), which may impact how they relate to professionals. Also, differences in roles within the family (e.g., earning the money or taking care of the children) may have led to different experiences, stresses and concerns when being forced to stay at home (Czymara et al., 2020).

## Method

### Participants & Procedure

The current study used routinely collected data of families starting an MST treatment in the Netherlands in the period between the 1st of June 2019 and the 31st of May 2020. Participating MST teams were operating in both urban and rural areas. Families completed monthly alliance assessments. As MST treatments have an average of three to five months, we included all assessments from T1 to T5.

Clients were informed that all data would be used for quality control and research purposes prior to starting treatment. As data collection was part of clinical practice and the data were provided anonymously to the researchers (i.e., it concerned retrospective file data) no further informed consent was required. The study complied with the American Psychological Association’s ethical principles regarding research with human participants.

Families were included in the study if they had at least one assessment between T1 and T5, resulting in a sample of 846 families, treated by 155 different therapists across 11 organizations. Families were referred to MST for severe externalizing behavioral problems of the adolescent (12–17 years). Adolescents with suicidal behavior, an autism spectrum disorder or who did not live at home with a caregiver could not participate in the intervention (MST Services, 2014). The mean age of the 846 participating adolescents was 14.60 years (SD = 2.00)^1^ and 63% were male. In the majority of the families, the primary caregiver was female (81%). A total of 125 families had at least one family member with an intellectual disability (from now on: families with an intellectual disability) and for 46 families concerns existed regarding child abuse or neglect (from now on: families with child abuse). The remainder of the families will be termed as regular families. Descriptive statistics are provided in Table 1.

**Table 1:** Descriptive statistics for full sample and subgroups

	Total sample (*n* = 846)		Regular families (*n* = 675)		Families with intellectual disability (*n* = 125)		Families with child abuse (*n* = 46)		Male caregivers (*n* = 160)		Female caregivers (*n* = 684)	
	*M*	*SD*	*M*	*SD*	*M*	*SD*	*M*	*SD*	*M*	*SD*	*M*	*SD*
Alliance T1	4.27	0.60	4.27	0.63	4.32	0.62	4.06	0.66	4.21	0.66	4.28	0.62
Alliance T2	4.44	0.59	4.42	0.60	4.59	0.47	4.19	0.70	4.38	0.68	4.45	0.56
Alliance T3	4.51	0.51	4.53	0.51	4.50	0.48	4.38	0.48	4.58	0.47	4.50	0.51
Alliance T4	4.51	0.53	4.51	0.54	4.57	0.43	4.34	0.56	4.56	0.44	4.50	0.55
Alliance T5	4.56	0.52	4.57	0.51	4.52	0.57	4.53	0.55	4.57	0.50	4.56	0.53

	No VC (*n* = 375)			Late transfer to VC (*n* = 189)				Early transfer to VC (*n* = 145)				Full VC (*n* = 137)			
	*M*	*SD*		*M*		*SD*		*M*		*SD*		*M*		*SD*	
Alliance T1	4.23	0.65		4.30		0.65		4.35		0.57		4.26		0.62	
Alliance T2	4.43	0.60		4.46		0.52		4.39		0.64		4.48		0.57	
Alliance T3	4.52	0.54		4.51		0.50		4.50		0.46		4.57		0.45	
Alliance T4	4.51	0.57		4.54		0.46		4.47		0.55		n/a		n/a	
Alliance T5	4.57	0.49		4.59		0.49		4.21		0.90		n/a		n/a	

In the Netherlands, a national lockdown due to the COVID-19 pandemic was issued on the 13th of March and lasted till the end of May, after which restrictions were gradually lifted. Although institutional policies regarding home visits and care delivery during the lockdown were not uniform, they all described that MST therapists primarily provided care through videoconferencing and that home visits were only conducted when this was deemed absolutely necessary and safe. This date has thus been used to categorize families in different lockdown groups, reflecting the moment at which the family started to use VC. We identified the following lockdown groups: (a) NO: families that did not have any alliance assessments during the lockdown (i.e., all assessments had taken place before the lockdown) (*n* = 375), (b) LATE: families that experienced the start of the lockdown late in treatment (after at least two months of treatment; *n* = 189), (c) EARLY: families that experienced the start of the lockdown early in treatment (within the first two months of treatment; *n* = 145) and (d) FULL: families who started treatment during the lockdown (*n* = 137).

### Measures

Alliance was measured using the Therapist Adherence Measure-Revised (TAM-R; Henggeler et al., 2006), a questionnaire of 28 items scored on a 5-point Likert scale (ranging from 1 = not at all to 5 = very much) and consisting of two scales named ‘Adherence’ and ‘Alliance’ (Lange et al., 2020). For this study, only the alliance scale was used, consisting of 11 items. An average score was calculated for each assessment if at least 7 of the 11 items were completed. These items tapped into the personal alliance (e.g. ‘My family and the therapist were honest and straightforward with each other’) as well as the task-related alliance (e.g. ‘Our family agreed with the therapist about the goals of treatment’). The TAM-R was completed on a monthly basis during treatment by the primary caregiver through a telephone interview. On average, 32% of the planned alliance assessments were not completed. Non-response was not dependent on lockdown, i.e., families were no more likely to miss an alliance assessment during the pandemic as prior to the pandemic.

### Statistical Analyses

We approached the research aim in two different ways. First, we ran Latent Growth Curve models (LGCM) to test whether the use of VC impacted upon the development of alliance during treatment. Second, we analyzed the impact of VC on the alliance level at several points in time during treatment using regression analyses. Families were nested within therapists (i.e., therapists treated multiple families within the sample), therapists were nested within teams, and teams were nested within organizations. The percentage of variance in alliance accounted for by these levels was 3%, 2% and 0% for the therapist, team and organization level, respectively. Although the variance accounted for by any one level was very low, in all analyses, we corrected for non-independence due to the nesting of families within therapists (see below for details), as alliance relates to a client-therapist relationship, making it theoretically relevant to include the therapist level in the models.

#### Latent Growth Curve Models

LGCMs were conducted in Mplus 7.4 (Muthén & Muthén, 1998–2015). Missing data were handled using robust full information maximum likelihood estimation. This approach makes use of all the available data and provides better estimations of standard errors when normality assumptions are violated. We accounted for the non-independence of the data due to therapists treating more than one family by adjusting the standard errors using the COMPLEX module in Mplus. First, we specified an LGCM for alliance for the whole sample, containing a fixed number of four time points (T1–T4), representing the first four months of the MST treatment.^2^ The fit of models with and without a quadratic slope was compared using the Akaike information criterion (AIC) and the Bayesian information criterion (BIC). As eleven families did not have a T1–T4 measurement (only a T5), these families were dropped from the LGCM analyses, resulting in a sample of 835 families.

We subsequently tested whether the trajectory of alliance was different for families experiencing the transition to VC at different points during their treatment (i.e., the lockdown groups as specified above; NO, LATE, EARLY and FULL). As many families in the full lockdown group had fewer than three assessments—the lockdown had lasted for 2.5 months—this group could not be analyzed separately and was therefore combined with the EARLY group for these analyses. A multi-group model was specified in which all parameters were estimated freely for each lockdown group. This model was compared with a more restrictive model in which the means of the intercept, linear slope and quadratic slope were restricted to be equal across the three lockdown groups. The Satorra and Bentler (2001) scaled chi-squared difference test was used to test whether the freely estimated model had a significantly better fit. A significant test would indicate that the freely estimated model, allowing for different trajectories across lockdown groups, fitted better.

To test the effect of the moderators, we added the moderators (type of family and gender of caregiver) as predictors of the intercept and growth factors in the multi-group analyses described above. We then compared the fit of a model in which the effects of the moderator were freely estimated across lockdown groups with the fit of a model in which these effect were constrained to be equal across lockdown groups.

#### Regression Analyses

We subsequently conducted a series of multilevel regression analyses with random intercepts and slopes in IBM SPSS Statistics 26 (IBM Corp. Released, 2019). The multilevel approach was chosen to account for the nesting of families within therapists. In these analyses, the alliance score was the dependent variable. Analyses were replicated for each of the five measurements (T1 to T5). In all analyses, we included the main effect of a variable representing whether the measurement had taken place during or prior to the lockdown. We also added the main effects of the possible moderators (type of family and gender of caregiver). Type of family was included as two dummy variables (regular vs. families with intellectual disability; regular vs. families with concerns around child abuse). In the second step, interaction effects between the lockdown variable and the moderators were added. The main effects allowed us to test whether using VC impacted upon the strength of the alliance and whether the moderators had any main effect on the alliance. The interaction effects allowed us to test whether the alliance was affected more strongly in certain populations than in others by using VC.

#### Effect of Full Lockdown

To zoom in on families that did not have any F2F contact at all (the FULL lockdown group), we replicated the regression analyses described above, including only families categorized in the NO (*n* = 375) and FULL (*n* = 137) lockdown groups. The lockdown variable now indicated whether treatment had taken place prior (NO) or during (FULL) the lockdown. These analyses were restricted to the T1 and T2 alliance measures, as the maximum duration of the lockdown for families was 2.5 months. Again, we added main effects in step 1 and interaction effects in step 2. To reduce the probability of Type 1 errors due to multiple tests, we used an alpha level of 0.01 for all regression analyses.

## Results

### Latent Growth Curve Models

Regarding the whole sample, model fit of an LGCM including both a linear and quadratic slope was better than the fit of a linear model (ΔAIC =  − 32; ΔBIC =  − 12). Fit of the quadratic model, based on the appropriate intercept-only null model (see Wu et al., 2009), was excellent (CFI = 1.00, TLI = 1.08, NFI = 1.00, χ^2^ = 0.23, *p* = 0.63). On average, alliance at the intercept was high (*M* [SE] = 4.25 [0.03], *p* < 0.001), increased during treatment (*M* [SE] = 0.22 [0.03], *p* < 0.001) and had a negative quadratic slope, pointing to a flattening curve over time (*M* [SE] =  − 0.05 [0.01], *p* < 0.01). The variances of the intercept (*p* = 0.00), linear (*p* = 0.01), and quadratic slope (*p* = 0.04) were also significant, indicating individual differences in initial levels and growth. Lastly, the covariances between the growth factors were significant. There was a positive covariance between the intercept and quadratic slope and a negative covariance between the linear slope and both the intercept and quadratic slopes (all *p*s < 0.05).

We subsequently compared a multi-group LGCM in which all parameters were freely estimated in the three lockdown groups to a model restricting the means of the intercept and growth factors to be equal across lockdown groups. The Satorra–Bentler chi-square difference test revealed no difference in model fit between the more restrictive and less restrictive model [ΔSB-χ^2^(6) = 3.53, *p* = 0.74]. Thus, the development of alliance during treatment was not different across the lockdown groups, meaning that the development of alliance was not associated to the timing of the VC.

To test for moderation by type of family and gender of caregiver, these variables were added as predictors of the intercept and growth factors. In the freely estimated multi-group model regarding type of family, there was one significant effect of child abuse on the intercept in the EARLY/FULL group (B [SE]_intercept_ = −0.32 [0.14], *p* = 0.02), meaning that families with child abuse who started treatment shortly prior or during lockdown reported a lower initial alliance than regular families. The multi-group model in which the effects of type of family were freely estimated across lockdown groups, however, did not fit significantly better [ΔSB-χ^2^ (12) = 13.98, *p* = 0.30] than the constrained model, indicating that overall the effects of type of family on the intercept and slope factors were not different across lockdown groups. In the constrained model, type of family did not have an effect on any of the growth factors (effect of intellectual disability: B [SE]_intercept_ = 0.09 [0.08], *p* = 0.26; B [SE]_linear slope_ = −0.05 [0.08], *p* = 0.56; B [SE]_quadratic slope_ = 0.01 [0.02], *p* = 0.76; Effect of child abuse: B [SE]_intercept_ = −0.17 [0.09], *p* = 0.06; B [SE]_linear slope_ =  − 0.04 [0.08], *p* = 0.64; B [SE]_quadratic slope_ = 0.01 [0.03], *p* = 0.67). The multi-group model in which the effects of gender were freely estimated across lockdown groups did not fit significantly better [ΔSB-χ^2^ (6) = 9.19, *p* = 0.16] than the model in which these effects were constrained to be equal across groups, indicating that the effects of gender on the intercept and slope factors were not different across lockdown groups. Gender did not have an effect on any of the growth factors (B [SE]_intercept_ = 0.07 [0.07], *p* = 0.30; B [SE]_linear slope_ =  − 0.08 [0.07], *p* = 0.24; B [SE]_quadratic slope_ = 0.01 [0.02], *p* = 0.74).

### Regression Analyses

**Fig. 1:**
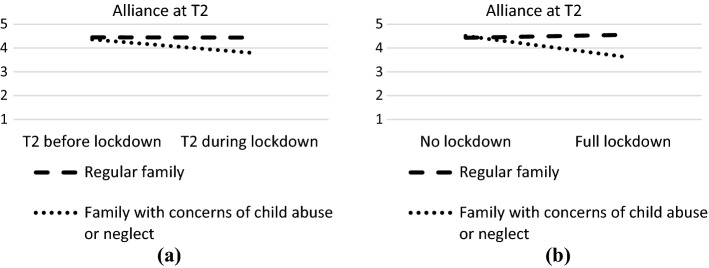
Interaction effect of lockdown

The results of the regression analyses assessing the effect of VC on alliance at the assessment moment are presented in Table 2. We found a significant main effect of ID at T2; families with ID reported a significantly higher alliance than regular families at T2 (*B* = 0.18, *p* = 0.03). We also found one significant interaction effect at T2 (see Fig. 1a). In families experiencing child abuse or neglect, VC was associated with lower levels of alliance at T2 (*B* =  − 0.57, *p* = 0.05), whereas in regular families, the effect of VC on alliance was nonsignificant (*B* = 0.01, *p* = 0.87). None of the other main or interaction effects were significant in any of the models, indicating that the lockdown and associated use of VC was not related to levels of alliance at T1, T3, T4 or T5. Also, we must note that none of the effects were significant when controlling for multiple testing.

**Table 2:** Unstandardized effects of regression analyses predicting alliance

Predictor	Alliance T1		Alliance T2		Alliance T3		Alliance T4		Alliance T5	
	Step 1	Step 2	Step 1	Step 2	Step 1	Step 2	Step 1	Step 2	Step 1	Step 2
*Main effects*										
Constant	4.20	4.18	4.37	4.29	4.59	4.58	4.57	4.53	4.61	4.60
Measurement in lockdown (yes = 1)	0.02	0.11	− 0.04	0.17	0.03	0.07	− 0.02	0.12	− 0.05	0.02
Type of family (regular—0 vs. intellectual disability—1)	0.06	0.08	0.18*	0.13	− 0.03	− 0.06	0.06	0.15	− 0.07	0.01
Type of family (regular—0 vs. child abuse—1)	− 0.19	− 0.03	− 0.21	− 0.06	− 0.14	− 0.06	− 0.18	− 0.10	− 0.05	0.10
Gender caregiver (female = 1)	0.07	0.10	0.08	0.18*	− 0.09	− 0.08	− 0.07	− 0.04	− 0.03	− 0.04
*Interaction effects*										
Lockdown x intellectual disability		− 0.07		0.13		0.09		− 0.27		− 0.23
Lockdown x child abuse		− 0.40		− 0.53*		− 0.26		− 0.28		− 0.42
Lockdown x Gender		− 0.08		− 0.26		− 0.05		− 0.10		− 0.02

*p < .05

### Effect of Full Lockdown

The results of the regression analyses focusing on families that had started treatment during lockdown are presented in Table 3. We found one significant main effect when predicting alliance at T2; at T2, women reported a higher alliance than men (*B* = 0.20, *p* = 0.03). We also found one significant interaction effect at T2; families experiencing child abuse or neglect reported a more negative impact of the lockdown and associated use of VC on their alliance than regular families (see Fig. 1b). In both types of families, however, the effect of VC on alliance was nonsignificant (*B* = 0.13, *p* = 0.15, in regular families; *B* =  − 0.39, *p* = 0.55, in families experiencing child abuse or neglect). None of the other main or interaction effects were significant in any of the models. Also, we must note that none of the effects were significant when controlling for multiple testing.

**Table 3:** Unstandardized effects of regression analyses for full lockdown predicting alliance

Predictor	Alliance T1		Alliance T2	
	Step 1	Step 2	Step 1	Step 2
*Main effects*				
Constant	4.15	4.08	4.25	4.21
Treatment in lockdown (yes = 1)	0.05	0.26	0.07	0.25
Type of family (regular—0 vs. intellectual disability—1)	0.04	0.04	0.19	0.14
Type of family (regular—0 vs. child abuse—1)	− 0.32	− 0.06	− 0.39	0.05
Gender caregiver (female = 1)	0.08	0.18	0.20*	0.26*
*Interaction effects*				
Lockdown x intellectual disability		− 0.04		− 0.15
Lockdown x child abuse		− 0.39		− 0.90*
Lockdown x Gender		− 0.24		− 0.21

*p < .05

## Discussion

This study investigated the impact of using VC during the COVID-19 pandemic on the alliance between therapist and primary caregivers in multi-stressed families receiving MST, using two different approaches. For most families, we found no evidence that the alliance was affected by using VC. Families experiencing child abuse or neglect reported lower levels of alliance when using VC at T2 (in the second month of treatment).

Similar to the adult psychotherapy literature and to one previous study on family-based therapy, we found that alliance is comparable in a VC and F2F setting (Berger, 2017; Comer et al., 2017; Simpson & Reid, 2014). This study extends previous findings by studying multi-stressed families and using a large sample. So far, little robust evidence was available for VC within this population. Moreover, this study was set during the COVID-19 pandemic which forced therapists to transition to VC. The current findings are especially encouraging as they suggest that a strong alliance can be build, even by therapists who may feel less experienced or less confident using VC and when the choice for VC was not based upon shared decision making.

We found some evidence that families experiencing child abuse or neglect reported a negative impact of VC on their alliance during the lockdown, especially if VC was used at the start of treatment. This finding was supported by the Dutch MST expert coaching therapists working with these families. She confirmed how difficult it is to engage these families through VC. As our sample was very small, and the results were no longer significant when controlling for multiple testing, the current results are only preliminary and suggestive. Replication is needed before we can draw any conclusions. So far, VC with high-risk populations has been understudied (De Boer et al., 2021). The current findings are of high clinical relevance as they urge us to further investigate the potential importance of F2F therapy to achieve engagement, motivation and alliance with these families. MST therapists working with these families tend to provide concrete support and visit families frequently to engage and motivate them (Kamphuis et al., 2015). This way of approaching and engaging families may be especially hard through VC, resulting in a lower alliance. Moreover, the COVID-19 pandemic has increased numerous stressors for families, such as parental unemployment, financial insecurity, increased burden of having to work while also home-schooling their children, and low levels of social support. These stressors are associated with parental stress and, subsequently, risks of child abuse (Griffith, 2020; Lawson et al., 2020). Although the evidence for an actual increase in child abuse during the pandemic is mixed (Blokhuis, 2020; Steketee et al., 2020), these increased stressors and risks for child abuse may have negatively impacted the alliance. Research has shown that symptoms and alliance mutually influence one another (Flückiger et al., 2020b). Thus, an increase in problems in the family due to the pandemic may have led families to feel less understood by their therapist, which may have resulted in families with concerns regarding child abuse or neglect to experience a lower quality working relationship.

We did not find any evidence for gender differences with regard to alliance when using VC or F2F in family therapy. So far, research into gender differences with regard to VC is sparse. One recent meta-analysis suggested that women may benefit more from VC than men as they found that women, but not men, receiving VC reported larger post-intervention changes than women receiving F2F (Batastini et al, 2021). Our study does not find a similar effect for reports of alliance.

In interpreting our findings, we bear in mind a number of limitations. First of all, although the national and institutional guidelines stated that sessions should be provided remotely, these guidelines did allow for F2F sessions in exceptional circumstances. Unfortunately, we have no data regarding which sessions were VC or F2F. This means that we must bear in mind that even families in the full lockdown group may have received some F2F sessions. Second, we only have data from families that continued treatment. It may be that families who experienced a deterioration of alliance dropped out of treatment or did not want to respond to the questionnaire. Indeed, communication with the Dutch MST purveyor organization showed that drop-out due to lack of engagement had increased in April and May to 9% (being 3% in the six months prior to the lockdown). This could indicate that a small percentage of families is more challenging to engage through VC. Alternatively, the higher drop-out percentage may be due to the COVID-19 pandemic itself rather than due to the use of VC. The pandemic did not impact upon the response-rate of families. Last, we only assessed alliance through the perspective of the primary caregiver, using a measure developed within the context of MST (Lange et al., 2020). Research suggests that therapists may be more negative about alliance through VC than clients (Berger, 2017; Norwood et al., 2018). Moreover, in family-based therapy, therapists need to balance alliances with all family members and a strong alliance with one member of the family may be detrimental for the therapeutic process if this leads to weak alliances with other members (i.e., a split alliance) (Friedlander et al., 2018; Muñiz de la Peña et al., 2009). Future research into VC for family-based interventions therefore needs to take all family members’ as well as therapists’ views into account.

This study also has several strengths. First, we included a large sample size, much larger than many prior studies, using routinely collected data. The results of this study therefore are a good representation of clinical practice. Second, we used two different approaches to our research questions, leading to similar results, meaning that our results are robust and can be interpreted with confidence. Third, we included more than 150 men and analyzed them separately. Men are underrepresented in child psychopathology research (Parent et al., 2018), as well as in therapeutic treatment of children’s mental health (Tully et al., 2017) and so far, no research on VC has looked into differential effects for men and women.

The results of this study are positive news for service providers as they show that most multi-stressed families develop strong alliances through VC. Future research will need to study whether VC also allows to attain positive treatment outcomes. Moreover, more research is needed to differentiate between families. This study provided preliminary evidence that VC may not be equally effective for everyone. More research in this direction is needed, allowing us to better understand whom might benefit more or less from VC and what tools therapists can use to improve VC delivery to those families that may struggle with this medium.

We must bear in mind that all families in this study received MST, an intervention with an elaborate quality assurance system and explicit attention to developing engagement and alliance with families throughout the whole treatment (Cunningham & Henggeler, 1999; Henggeler et al., 2009). Moreover, all MST teams were supported from the onset through consultation and documentation on how to respond to the COVID-19 pandemic, including best practices and tips and strategies. These qualities of MST have most likely supported therapists to develop and maintain strong alliances during such challenging times. These results therefore do not necessarily generalize to other family-based interventions. We need to critically appraise and compare contexts before generalizing these results to other situations.

The current study only studied the first two to three months of the pandemic. Although restrictions were lifted in May, VC has continued to be used to some extent; for example when family members or the therapist had to self-isolate or when new restrictions were put in place following increasing COVID-19 cases. This pandemic, therefore, provides a unique opportunity and experimental situation to study VC and other forms of telepsychotherapy. We can only but encourage researchers to seize this and similar future opportunities to study treatment aspects such as alliance, adherence, engagement and motivation, drop-out and treatment outcomes in a VC setting. Moreover, we need to interview therapists and families to hear about their experiences and use all this information to benefit the future. This information can be used to develop VC treatments or blended therapies, for example to reach populations in remote areas that may not have access to evidence-based interventions otherwise, or to develop more personalized care.

## Conclusions

This study suggests that caregivers in multi-stressed families receiving family-based therapy develop and maintain strong alliances with their therapist during VC sessions, which are comparable to alliances in F2F sessions. This is positive support for the use of VC with these families and is a compliment to all therapists who have persevered during challenging times. Nevertheless, more research is needed to better understand the boundaries of using VC for family-based therapy. For example, the current study suggests that building strong alliances with families with concerns around child abuse or neglect is more challenging through VC. We look forward to future research and clinical initiatives to explore the possibilities of VC for family-based therapy.

## Data Availability

The data is owned by the health care intuitions and is anonymously stored at the Tilburg University Dataverse and metadata will be stored at the Health Ri portal. Interested researchers can ask permission by the authors to get access to the data. This may involve requesting permissions of the institutions.

## Footnotes

1 The mean age for families treated regarding concerns around child abuse was 11.70 (SD = 3.12), ranging from 5 to 17 years, as the MST inclusion criteria regarding age is larger for these families.

2 Specifying a model with five time points led to low covariance coverage in the multiple group analyses; families having experienced the transition to VC early in treatment were not likely to have finished treatment by the end of the data collection period and therefore did not have five assessments.

## Code availability

SPSS syntax and Mplus code is available upon request.
